# Videothoracoscopic lobectomy: first year of experience in a single center

**DOI:** 10.1186/1749-8090-8-S1-O223

**Published:** 2013-09-11

**Authors:** A Gonfiotti, D Barale, MO Jaus, F Montinaro, F Mannini

**Affiliations:** 1 Thoracic Surgery Division, Careggi University Hospital, Florence, Italy

## Background

The purpose of this work is to evaluate the feasibility, safety and oncological appropriateness of a completely videothoracoscopic surgical program of major pulmonary resections (VATS-L) in a single center, in its first year of development.

## Methods

From April 2012 to date, we performed 42 completely thoracoscopic lobectomies (non rib-spreading) with anterior approach to the pulmonary hilum. Clinical stages I were included and T3 or T4 tumors, central tumors, cN1-N2, previous ipsilateral thoracotomy were excluded. Whenever possible, we have obtained a preoperative diagnosis with CT-guided needle biopsy (n = 35, 83%); otherwise lobectomy was preceded by atypical resection and extemporaneous examination. A preoperative mediastinoscopy was performed following the guidelines of the National Comprehensive Cancer Network, 2013 (n = 18, 43%), in the same operative time with extemporaneous examination of the lymph nodes. Lung resection was always associated with a lymphadenectomy of at least 4 ilo-mediastinal stations.

## Results

we treated 22 females and 20 males (mean age 71 years, range 54-82) in which we performed: right upper lobectomy n = 10, n = 12, left upper lobectomy, middle lobectomy n = 4, right lower lobectomy n = 9 and left lower lobectomy n = 7. The main number of lymph nodes taken was 5.8 (range: 4-9). In 4 cases (9%) it was necessary to convert the procedure, in 3 cases for minor arterial lesions and in one case for venous injury. The mean operative time was 150 minutes (range 90-270). The histopathologic examination revealed a N1 disease in 5 cases (12%) and in 1 case N2 (2%). The main time of hospitalization was 5 days (range: 3-8). We did not record cases of 30-day mortality.

## Conclusions

On the basis of the results of this first year of experience in a single center, VATS-L appears to be a safe and effective alternative to traditional surgery even in the initial phase of a program of development of this technique.

